# Lymphocytic choriomeningitis virus meningitis after needlestick injury: a case report

**DOI:** 10.1186/s13756-019-0524-4

**Published:** 2019-05-20

**Authors:** Sarah Dräger, Anna-Friederike Marx, Fiona Pigny, Pascal Cherpillod, Philip Eisermann, Parham Sendi, Andreas F. Widmer

**Affiliations:** 1 Infectious Diseases and Hospital Epidemiology, University Hospital Basel, Petersgraben 4, 4031 Basel, Switzerland; 2 Department of Biomedicine – Haus Petersplatz, Division of Experimental Virology, University of Basel, 4009 Basel, Switzerland; 3 Laboratory of Virology, Department of Genetic and Laboratory Medicine, University Hospitals of Geneva, Rue Gabrielle-Perret-Gentil 4, 1211, 14 Geneva, Switzerland; 4 WHO Collaborating Centre for Arbovirus and Haemorrhagic Fever Reference and Research, Bernhard Nocht Institute for Tropical Medicine, Bernhard-Nocht-Strasse 74, 20359 Hamburg, Germany; 5 Institute for Infectious Diseases, University of Bern, Freiburgstrasse 18, 3010 Bern, Switzerland

**Keywords:** Lymphocytic choriomeningitis virus, Meningitis, Needlestick injury, Accidental infection, RT-PCR

## Abstract

**Background:**

Needlestick accidents while handling of infectious material in research laboratories can lead to life-threatening infections in laboratory personnel. In laboratories working with the lymphocytic choriomeningitis virus (LCMV), the virus can be transmitted to humans through needlestick injury and lead to serious acute illness up to meningitis.

**Case presentation:**

We report of a case of LCMV meningitis in a laboratory worker who sustained a penetrating needlestick injury with a LCMV-contaminated hollow needle whilst disposing of a used syringe into the sharps waste bin. Four days after needlestick injury the laboratory worker developed a systemic disease: 11 days after exposure, she was diagnosed with meningitis with clinical signs and symptoms of meningismus, photophobia, nausea and vomiting, requiring hospitalisation. The PCR was positive for LCMV from the blood sample. 18 days after exposure, seroconversion confirmed the diagnosis of LCMV-induced meningitis with an increase in specific LCMV-IgM antibodies to 1:10′240 (day 42: 1:20′480). Ten weeks after exposure, a follow-up titre for IgM returned negative, whereas IgG titre increased to 1:20′480.

**Conclusions:**

This is the first case report of a PCR-documented LCMV meningitis, coupled with seroconversion, following needlestick injury. It highlights the importance of infection prevention practices that comprise particularly well established safety precaution protocols in research laboratories handling this pathogenic virus, because exposure to even a small amount of LCMV can lead to a severe, life-threatening infection.

**Electronic supplementary material:**

The online version of this article (10.1186/s13756-019-0524-4) contains supplementary material, which is available to authorized users.

## Background

LCMV infection is usually asymptomatic or mild and self-limiting, but rarely can manifest itself as severe disease such as meningitis and encephalitis [1]. In organ transplant recipients and immunocompromised patients such an infection can be life-threatening [2, 3]. Congenital infections can result in life-long mental retardation and vision deficits [4]. House mice (*Mus musculus*) are the common host of LCMV, a member of the family of *Arenaviridae*. Modes of transmission to humans are bites of infected mice, inhalation of aerosolized droplets of contaminated body fluids or inoculation of contaminated materials into broken skin, the eyes or the mouth [5].

We report of well-documented LCMV-meningitis of a laboratory worker after needlestick injury confirmed by serological tests and direct detection of viral RNA in blood by PCR. A previous case report of lab-acquired LCMV infection was based on indirect evidence by serological tests, not direct detection of viral RNA by PCR [6]. There is no evidence-based standard drug available for post-exposure prophylaxis: Therefore, prevention of stab wounds with contaminated sharp objects, bite of infected mice and inhalation of contagious droplets, is indispensable to protect laboratory worker from occupational severe infection, leading even to epidemics [7].

This highlights the importance of infect prevention strategies that comprise particularly well established safety precaution protocols in research laboratories handling this pathogenic virus, because exposure to LCMV can lead to a life-threatening infection.

## Case presentation

In May 2017, a young scientist accidently sustained a penetrating needlestick injury to the left index finger from a LCMV-contaminated needle, whilst disposing of a used syringe in a correct manner into the inaccurate overfilled sharps waste bin. The needle stuck on a syringe that was used to infect mice with Lymphocytic choriomeningitis virus (LCMV) variant clone-13. For its distinct use, the syringe was filled up with 200 μL fluid solution containing a LCMV-concentration of 9.15 × 10^6^ Plaque-forming units (PFU)/ml and was littered after its use, presumably completely emptied. The estimated inoculated volume was approximately 1 μL (corresponding to 9.15 × 10^3^ PFU). Following strictly the laboratory safety protocol, first aid measures were taken immediately after the injury by washing and disinfecting the wound. A check-up at the university emergency room (ER) the same day, with clinical examination and blood tests remained unremarkable. As there is no known well-established medicamentous post-exposure prophylaxis, the patient was discharged and was advised to return to the ER if any signs of illness occur.

**Fig. 1:**
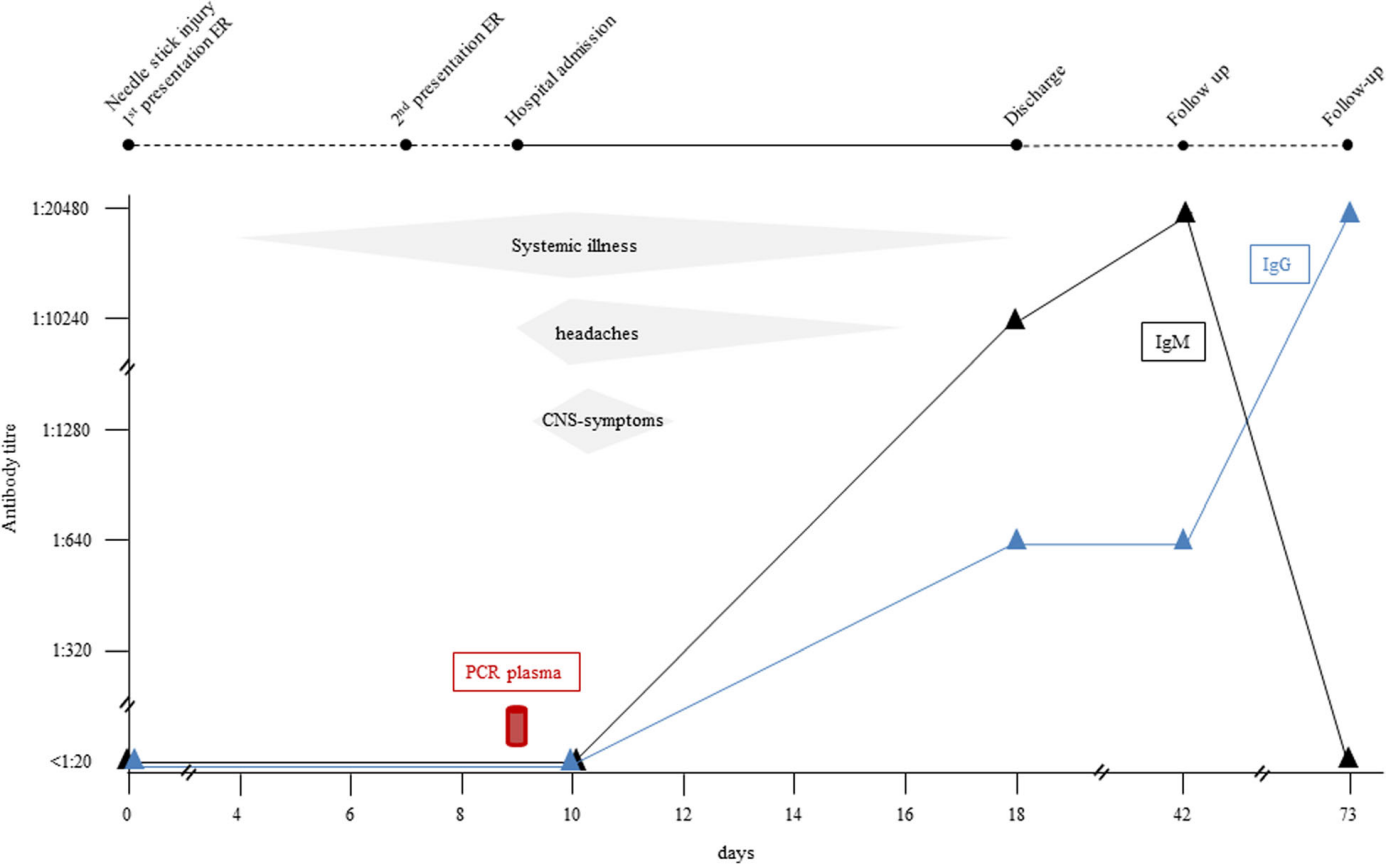
Clinical presentation, symptoms and serological tests over the course of time since the day of needlestick injury (Day 0). On day nine: detection of LCMV-RNA in the blood plasma by PCR. CNS-symptoms include meningismus, photophobia, nausea and vomiting. (ER: emergency room)

Four days after needlestick injury, she developed systemic illness, comprising acute severe lower back pain and fatigue (Fig. 1).

Seven days after exposure, her clinical situation deteriorated by developing flu-like illness with cough, pharyngitis, neck pain, fever up to 38.3 °C, chills and pain in the limbs. She presented to the ER where physical examination revealed no signs of meningitis or focal neurological deficits. Blood tests showed acute leukopenia (2.1 × 10^9^/l) and lymphopenia (0.57 × 10^9^/l) (Additional file 1: Figure S1), but C-reactive protein and liver function tests were within the normal range (data not shown). She was discharged with symptomatic, pain-relieving therapy.

Nine days after needlestick injury, symptoms of systemic illness, especially headaches and back pain, relapsed and worsened over time. She was admitted to the hospital where supportive care measures - rehydration and intensified pain relief - were established. Cerebrospinal fluid (CSF) showed cell count in the normal range as well as protein, glucose and lactate level. Serological tests were reactive for tick-borne-encephalitis (IgG and IgM positive) consistent with her known positive vaccine status. A multiplex polymerase chain reaction (PCR) performed in the CSF, including six bacterial species, seven viruses and one yeast, remained negative. MRI of the lumbar spine and the brain showed no evidence for abscess or meningeal enhancement. Thrombocytes dropped from 156 × 10^9^/l to 92 × 10^9^/l and lymphocytes from 0.57 × 10^9^/l to 0.36 × 10^9^/l, consistent with onset of acute viral infection (Additional file 1: Figure S1).

11 days after exposure, she was diagnosed with meningitis, with clinical signs and symptoms of meningismus, photophobia, nausea and vomiting, suggesting acute LCMV-meningitis.

Blood plasma samples that were drawn nine days after injury were simultaneously sent to the Laboratory of Virology at the University Hospital of Geneva and to the Bernhard Nocht Institute for Tropical Medicine in Hamburg for LCMV-PCR. The Geneva laboratory performed an LCMV-specific real-time reverse transcriptase (RT)-PCR assay [8] and reported a positive PCR signal for LCMV from the blood sample. Although CT-Value was high, indicating a low viral load (CT Mean 40.868), the signal was clearly positive. The other reference laboratory in Hamburg obtained a negative result using a conventional RT-PCR assay [9]. LCMV-PCR performed in two urine samples (day 14 and 15 after exposure) and in the CSF (day nine after exposure) remained negative, reported by the Geneva laboratory. Serological testing was performed in Hamburg by immunofluorescence assays using LCMV-infected cells as antigen. At day of the needlestick injury and ten days after exposure, the patient was tested negative for LCMV-specific IgM as well as IgG (Fig. 1).

12 days after exposure the patient improved, and laboratory findings returned to normal ranges.

18 days after exposure, seroconversion with an increase in specific LCMV-IgM antibodies to 1:10′240 (day 42: 1:20′480; negative reference < 1:20) was confirmed (s. Figure 1). Already to that point of time, an increase in specific LCMV-IgG-antibodies to 1:640 could be noted (day 42: 1:640; negative reference < 1:20) (s. Figure 1). Ten weeks after exposure, a follow-up titre for IgM returned negative, whereas IgG titre increased up to 1:20′480 (s. Figure 1). Diagnosis of acute LCMV meningitis after needlestick injury was based on epidemiological links and exposure, clinical signs and symptoms, seroconversion in plasma and detection of virus RNA in blood plasma by PCR. A diagnostic work-up at the clinic of neurology did not find any evidence for an alternative diagnosis.

## Discussion and conclusion

House mice are the common host of LCMV, a member of the family of *Arenaviridae*. Modes of transmission to humans are bites of infected rodents, inhalation of aerosolized droplets of contaminated body fluids or inoculation of contaminated materials into broken skin, the eyes or the mouth [5]. LCMV infection is usually asymptomatic or mild and self-limiting, but rarely can manifest itself as meningitis and encephalitis [1]. In organ transplant recipients and immunocompromised patients, such infections can be life-threatening [2, 3]. Congenital infections can result in life-long mental retardation and vision deficits [4]. The incubation period usually is eight to 13 days followed typically by a biphasic febrile illness [10]. The initial phase begins with fever, malaise, muscle aches, headache and nausea and is lasting for about one week [10]. After a few days of recovery signs of meningoencephalitis occur [10]. Our patient developed this typical biphasic illness, though it already occurred four days after exposure. The cause of the shorter incubation period is most likely the high inoculum with which the patient has become infected and the mode of transmission via direct inoculation in the skin.

It is of note that even in patients presenting with signs and symptoms of meningitis, CSF cell count still can be in the normal range as well as protein, glucose and lactate level.

We assume that the conventional RT-PCR that was used in Hamburg might be less sensitive than the LCMV-specific real-time RT-PCR performed in Geneva leading to the differing results. In vitro, detection limit of RT-PCR is 20 PFU/mL, whereas RNA-detection of real-time RT-PCR can succeed with viral amounts of ≤10 PFU/mL [8, 9]. PCR-signal obtained with real-time RT-PCR was weak but clearly positive (CT Mean 40.868), indicating a very low viral load.

This is the first published case report of well-documented LCMV-meningitis after needlestick injury confirmed by serological tests and direct detection of viral RNA in blood by PCR. A previous case report of iatrogenic LCMV infection was based on indirect evidence by serological tests, not direct virus detection by PCR, although the inoculum was presumably significantly higher than in our case [6]. There is no evidence-based standard drug available for post-exposure prophylaxis. The role of ribavirin in LCMV infection remains unclear. In mice, an effectiveness of ribavirin has been demonstrated, but this effect remains unclear in humans [2]. Prevention of stab wounds with contaminated sharp objects, bite of infected mice and inhalation of contagious droplets, is crucial to protect laboratory worker from iatrogenic infection, leading even to epidemics [7]. LCMV-outbreaks in healthcare workers [11] and laboratory personnel [12, 13] are well described. The source of illness for these outbreaks are rodents that have been infected with LCMV for research purposes. LCMV then was transmitted to humans either via direct intimate contact with LCMV-infected rodents [12, 13] or by indirect contact via aerosol exposure demonstrating the risk for transmission [11, 13]. Therefore, laboratory personnel must be instructed of the possible transmission routes and clinical presentation of LCMV with repetitive instructions on safe handling of LCMV. When working with LCMV, high quality gloves, goggles and protective gowns must be worn, as well as appropriate ventilation systems should be in place where animals are handled. Surfaces should be routinely disinfected using a certified compound active against LCMV. In addition, a standard operating procedure should be available for the management of unintentional exposure to the virus. Pregnant women and immunosuppressed persons must be taught that exposure to even a small amount of this pathogenic virus can lead to a severe, life-threatening infection.

In conclusion, this well-documented case of LCMV-meningitis after needlestick injury highlights the importance of infection prevention practices in research laboratories and the need of safe handling of this virus, whose pathogenicity often is underestimated.

## Additional file

Additional file 1: **Figure S1**: Thrombocytes, neutrophils and lymphocytes over the course of time after day of needlestick injury. (TIF 27480 kb)

## Abbreviations

CSF: Cerebrospinal fluid
ER: Emergency room
LCMV: Lymphocytic choriomeningitis virus
PCR: Polymerase chain reaction
PFU: Plaque-forming unit
RT: Reverse transcriptase
