# Correction to: Lymphocytic choriomeningitis virus meningitis after needlestick injury: a case report

**DOI:** 10.1186/s13756-019-0600-9

**Published:** 2019-08-22

**Authors:** Sarah Dräger, Anna-Friederike Marx, Fiona Pigny, Pascal Cherpillod, Philip Eisermann, Parham Sendi, Andreas F. Widmer

**Affiliations:** 1 Infectious Diseases and Hospital Epidemiology, University Hospital Basel, Petersgraben 4, 4031 Basel, Switzerland; 2 Department of Biomedicine–Haus Petersplatz, Division of Experimental Virology, University of Basel, 4009 Basel, Switzerland; 3 Laboratory of Virology, Department of Genetic and Laboratory Medicine, University Hospitals of Geneva, Rue Gabrielle-Perret-Gentil 4, 1211 14 Geneva, Switzerland; 4 WHO Collaborating Centre for Arbovirus and Haemorrhagic Fever Reference and Research, Bernhard Nocht Institute for Tropical Medicine, Bernhard-Nocht-Strasse 74, 20359 Hamburg, Germany; 5 Institute for Infectious Diseases, University of Bern, Freiburgstrasse 18, 3010 Bern, Switzerland

**Correction to: Antimicrob Resist Infect Control (2019) 8:77**

**https://doi.org/10.1186/s13756-019-0524-4**

The original article [1] contains several mentions of a potentially misleading assertion which the authors would like to clarify; each mention relating to LCMV needlestick injury and its potential capacity for injury.

As such, the authors would like to clarify that in the context of this manuscript, LCMV needlestick injuries may only lead to life-threatening infections in immunosuppressed persons.

The affected statements in the manuscript and their corrected version can be viewed ahead:

1. The Conclusions sub-section of the Abstract should instead state:
  “This is the first case report of a PCR-documented LCMV meningitis, coupled with seroconversion, following needlestick injury. It highlights the importance of infection prevention practices that comprise particularly well established safety precaution protocols in research laboratories handling this pathogenic virus, because exposure to LCMV can lead to a severe infection.”
2. The latter part of the penultimate paragraph of the Background section should instead state:
  “Therefore, prevention of stab wounds with contaminated sharp objects, bite of infected mice and inhalation of contagious droplets, is indispensable to protect laboratory worker from occupational infection.”
3. The final paragraph of the Background section should instead state:
  “This highlights the importance of infect prevention strategies that comprise particularly well established safety precaution protocols in research laboratories handling this pathogenic virus, because exposure to LCMV can lead to a severe infection.”
4. A middle sentence in the Discussion and conclusion section should instead only state:
  “Prevention of stab wounds with contaminated sharp objects, bite of infected mice and inhalation of contagious droplets, is crucial to protect laboratory worker from iatrogenic infection.”
5. The latter half of the penultimate paragraph of the Discussion and conclusion section should instead state the following:
  “When working with LCMV in research laboratories, standard biosafety 2 laboratory practices are mandatory. This includes an appropriate ventilation system as well as wearing high quality gloves and protective gowns when working with LCMV. Surfaces should be routinely disinfected using a registered compound active against LCMV. In addition, a standard operating procedure should be available for the management of unintentional exposure to the virus. Pregnant women and immunosuppressed persons must be taught that exposure to this virus can lead to a severe, life-threatening infection.”
6. The final paragraph of the Discussion and conclusion section should instead only state:
  “In conclusion, this well-documented case of LCMV-meningitis after needlestick injury highlights the importance of infection prevention practices in research laboratories and the need of safe handling of this virus.”
