# Supplementary figures and images for: Lymphocytic choriomeningitis virus meningitis after needlestick injury: a case report
Source: Antimicrob Resist Infect Control. 2019 May 20;8:77. doi: 10.1186/s13756-019-0524-4 (PMC6528361; doi:10.1186/s13756-019-0524-4)

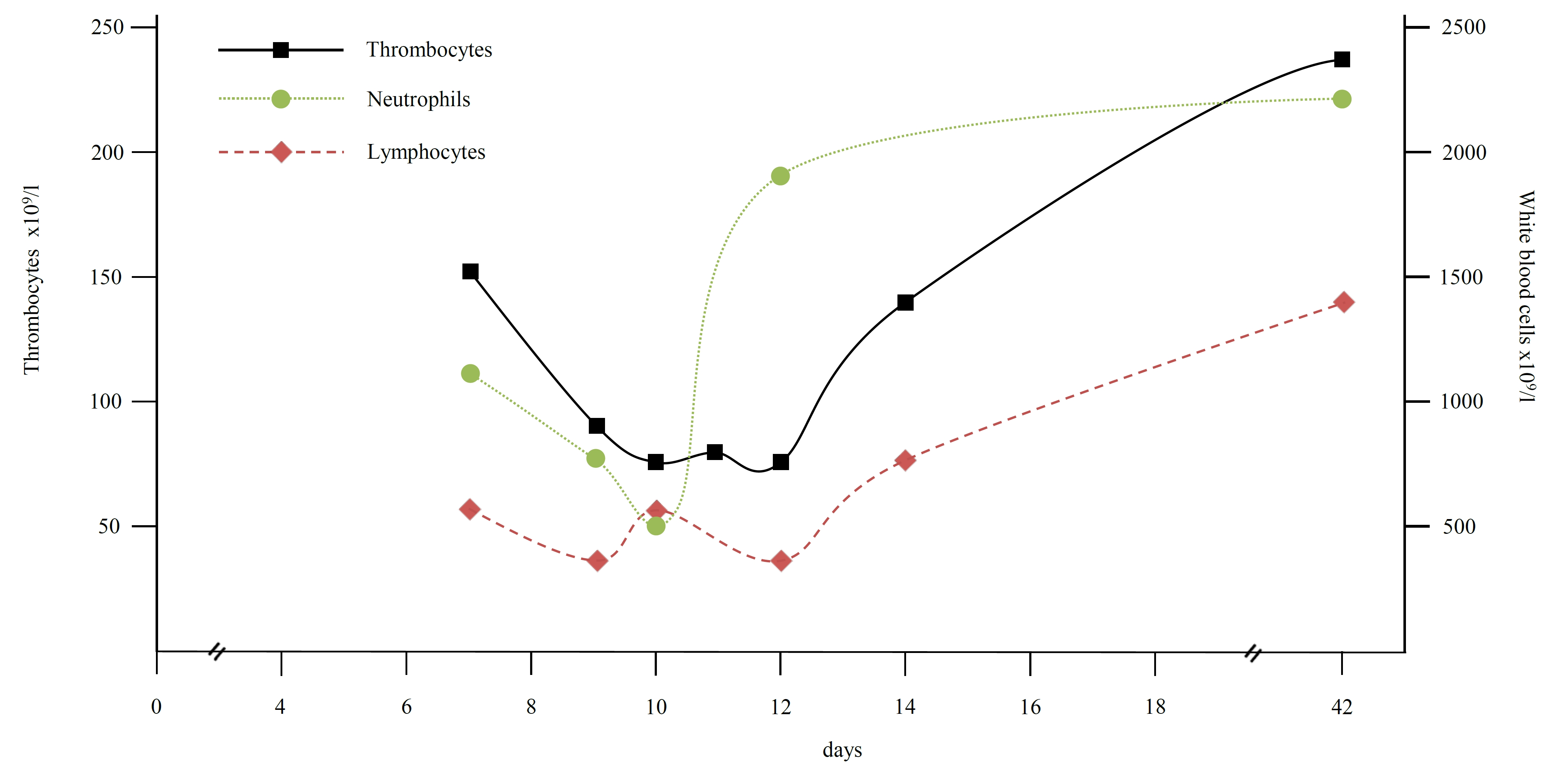

Supplement: Supplementary file 1 — Figure S1: Thrombocytes, neutrophils and lymphocytes over the course of time after day of needlestick injury. (TIF 27480 kb) [file 13756_2019_524_MOESM1_ESM.tif]
